# Digital twin technology for HIV patient virtual modeling: a novel approach to treatment optimization

**DOI:** 10.3389/fphar.2026.1836330

**Published:** 2026-08-28

**Authors:** Yuan Zhang, Tingting Li, Jie Chen, Huanhuan Ba, Jiajia Li, Jinling Yin, Kangxiao Ma, Huanqing Liu, Juan Jin

**Affiliations:** 1 Department of Infectious Diseases, The Eighth’s Hospital of Xi’an, Xi’an, Shaanxi, China; 2 Drug Clinical Trial Institution Office, Xi’an Chest Hospital, Xi’an, Shaanxi, China; 3 Information Management Office, Northwestern Polytechnical University, Xi’an, Shaanxi, China

**Keywords:** digital twin, HIV, machine learning approaches, personalized treatment, precision medicine, treatment optimization

## Abstract

**Background:**

Human immunodeficiency virus (HIV) infection remains a global health challenge, necessitating personalized treatment strategies to optimize patient outcomes. Digital twin technology—synchronized with real-world entities—offers a pathway to precision medicine in HIV care.

**Methods:**

We analyzed 5,436 HIV patients (2016–2024) with demographic, immunologic, and antiretroviral therapy (ART) information. Core algorithms comprised gradient boosting and random forest ensembles; deep architectures (LSTM and Transformer networks) were evaluated as comparative benchmarks on longitudinal feature tensors under identical supervision. Models used five-fold cross-validation, regularization, dropout, and early stopping. A probabilistic treatment ranking layer generated diversified regimen suggestions conditioned on model outputs.

**Results:**

Ensemble models achieved strong explained variance on the registry-defined CD4 label (test R^2^ up to 0.97; cross-validation stability reported in the Results). Deep models achieved comparable held-out accuracy on the same endpoint; the ranking module produced balanced regimen distributions in an illustrative optimization cohort. Simulated digital twin scenarios demonstrated counterfactual CD4 projections under alternative ART strategies for low, moderate, and higher baseline immunology profiles.

**Discussion:**

The specific contribution is an explicit patient-state → prediction → regimen exploration pipeline suited to integration with telehealth analytics (risk dashboards, adherence supports), while transparently acknowledging endpoint and leakage limitations and the need for prospective outcome validation. A pragmatic HIV treatment optimization strategy is to route model-supported prioritization into clinician-facing dashboards and remote monitoring workflows rather than autonomous prescribing.

## Introduction

Human immunodeficiency virus (HIV) infection remains a major global health burden, with an estimated 38 million individuals living with the virus as of 2023 (UNAIDS, 2024). Although the advent of antiretroviral therapy (ART) has transformed HIV from a once-fatal disease into a manageable chronic condition, achieving optimal treatment outcomes continues to be challenged by substantial inter-individual variability in drug efficacy, adherence patterns, and disease progression (Altice et al., 2019). This clinical complexity underscores the pressing need for personalized treatment strategies that integrate patient-specific factors, prior therapeutic history, and predicted clinical trajectories to improve long-term outcomes (Sah et al., 2025).

Digital twin technology, originally developed for industrial applications, has recently emerged as a transformative paradigm in healthcare (Corral-Acero et al., 2020). Recent work has explicitly extended digital twin concepts to HIV care, proposing a multi-layered framework incorporating virological, pharmacological, behavioral, and demographic layers to create a continuously learning computational representation of the individual (Siddiqui and Khan, 2026). A digital twin is a virtual representation of a physical entity that mirrors its real-world counterpart through continuous data integration and dynamic modeling (Björnsson et al., 2019). In the clinical context, digital twins enable the simulation of patient states, prediction of treatment responses, and optimization of therapeutic interventions prior to real-world implementation (Laubenbacher et al., 2021). This shift from reactive to predictive medicine holds significant promise for advancing the management of chronic diseases, including HIV.

Recent advances in artificial intelligence, particularly deep learning, have revolutionized predictive modeling in healthcare. Long Short-Term Memory (LSTM) networks excel at capturing temporal dependencies in longitudinal patient data, making them well-suited for modeling disease trajectories and treatment responses over time (Rajkomar et al., 2019). Transformer architectures, leveraging attention mechanisms, have further advanced sequence modeling by enabling models to dynamically focus on the most clinically relevant features, thereby enhancing both predictive accuracy and interpretability (Moor et al., 2023). These complementary capabilities provide a robust computational foundation for developing personalized HIV management strategies. In HIV research, machine learning has been applied to predict diverse outcomes, including immunological non-response to ART, with recent multicenter studies in China demonstrating strong predictive performance (AUC up to 0.864) (Sun et al., 2026). However, these approaches typically focus on isolated endpoints such as immune non-response or viral suppression, without linking predictions to an actionable optimization framework that simulates patient-specific outcomes under alternative regimens.

Despite methodological progress in HIV prediction modeling, a critical research gap remains: few studies have integrated ensemble learners, sequence-based architectures (e.g., LSTM, Transformer), and an optimization shell within a single, digitally articulated workflow. Even fewer have aligned such modeling outputs with operational pathways such as telehealth monitoring or clinician dashboards. Prior HIV machine learning work has largely focused on isolated prediction tasks—such as predicting virological failure, CD4 decline, or treatment retention—without linking these predictions to actionable regimen exploration under clinical uncertainty (Marcus et al., 2020). This disconnect between predictive modeling and decision support represents a major barrier to the clinical adoption of artificial intelligence in HIV care.

In this study, we described an application of a digital twin framework for virtual modeling of HIV patients, integrating multiple advanced machine learning approaches. Our system addresses three critical challenges: (1) accurate prediction of individual treatment responses using ensemble methods, (2) optimization of personalized treatment regimens through multi-objective optimization, and (3) simulation of long-term clinical outcomes under alternative therapeutic scenarios. Leveraging a large-scale clinical dataset comprising 5,436 HIV patients with detailed clinical and demographic information, we demonstrate the feasibility and effectiveness of digital twin technology in HIV precision medicine. This work contributes to the growing field of digital health by offering a scalable framework for patient virtual modeling, treatment optimization, and clinical decision support. Our approach has the potential to improve patient outcomes, reduce healthcare costs, and advance precision medicine for HIV and other chronic diseases. The integration of multiple machine learning architectures ensures robust predictive performance while preserving clinical interpretability through feature importance analysis and attention mechanisms.

## Methods

### Data collection, preprocessing and eligibility criteria

We analyzed a comprehensive dataset comprising 5,436 HIV patients from a major healthcare institution, with data collected between 2016 and 2024. The dataset was obtained from Xi’an Eighth Hospital (Xi’an, Shaanxi Province, China, 710061), a major tertiary care center specializing in infectious diseases. The dataset encompassed demographic information (age, gender, ethnicity, occupation, education level), clinical measurements (CD4 count, CD8 count, CD4/CD8 ratio, body mass index, blood pressure, heart rate), treatment regimens, and temporal variables (date of diagnosis, antiretroviral therapy initiation date, follow-up visit dates). The primary outcome variable for prediction was the baseline CD4 count, a key immunological marker that informs treatment decisions and prognostic assessment. The study protocol was reviewed and approved by the Institutional Review Board of Xi’an Eighth’s Hospital (approval no. 20250811), which waived the requirement for individual informed consent due to the retrospective, anonymized nature of the data.

### Eligibility criteria

Inclusion criteria were: (1) laboratory-confirmed diagnosis of HIV-1 infection; (2) initiation of a standard ART regimen within the defined study period; (3) availability of baseline CD4^+^ and CD8^+^ T-cell counts measured within 3 months prior to ART initiation (these baseline values were used solely for cohort definition and descriptive reporting, and were excluded from model inputs); and (4) at least one documented follow-up clinical visit with relevant laboratory data post-ART initiation.

Exclusion criteria included: (1) incomplete baseline demographic or clinical records; (2) missing values for key predictor variables (e.g., baseline viral load, core clinical parameters); and (3) records with unresolved data quality issues or logical inconsistencies (e.g., implausible laboratory values).

### Missing data handling and uncertainty assessment

We assumed a predominantly Missing At Random (MAR) mechanism conditional on observed covariates for retained fields, while acknowledging potential Missing Not At Random (MNAR) patterns when testing intensity correlates with illness severity (e.g., vital-sign gaps among non-attenders) (Sun et al., 2026). Missing numeric values were handled using multiple imputation by chained equations (MICE) with 20 imputed datasets and 20 iterations each, using predictive mean matching for continuous variables. Categorical fields were imputed using mode imputation within the MICE framework. Imputation preceded z-score scaling. To propagate imputation uncertainty, we compared three strategies on identical train/validation/test splits: (1) primary MICE (replacing the previously considered median/mode approach), (2) complete-case analysis requiring non-missing values across all retained predictors, and (3) MICE plus binary missingness indicators for fields with >5% missing (Table 1). Table 2 summarized the highest-burden missing fields among variables still present in the registry.

**TABLE 1:** Sensitivity of CD4 prediction performance to missing-data handling strategies.

Missing-data strategy	Model	n	Test R^2^	Test RMSE (cells/μL)
Primary (MICE)	Gradient boosting	5,436	0.974	34.3
Primary (MICE)	Random forest	5,436	0.918	61.1
Complete-case analysis	Gradient boosting	4,628	0.925	64.8
Complete-case analysis	Random forest	4,628	0.852	94.9
MICE + missing indicators	Gradient boosting	5,415	0.974	34.4
MICE + missing indicators	Random forest	5,415	0.917	61.5

**TABLE 2:** Missing data prevalence among registry fields with non-zero missingness (excluding 100% missing fields removed before modeling).

Variable	Missing, n	Missing, %
Alcohol amount	5,240	96.4
Smoking amount	4,813	88.5
Heart rate	806	14.8
Systolic blood pressure	697	12.8
Diastolic blood pressure	693	12.7
Non-AIDS comorbidity flag	587	10.8
Alcohol use	557	10.2
Smoking status	550	10.1

Features were standardized using z-score normalization (van der Heijden et al., 2006). Categorical variables were label-encoded where appropriate. Outliers were Winsorized using the interquartile range (IQR) method to limit undue leverage (van der Heijden et al., 2006).

Note on standardization and propensity matching. CD4 and CD8 counts are absolute values (cells/μL) and are not adjusted for body weight or BMI, as no weight-based normalization is clinically indicated for these lymphocyte subsets. We therefore used z-score standardization to bring all continuous variables (including CD4, CD8, age, BMI, blood pressure) onto a common scale. Propensity score matching was not performed; all analyses used the full cohort with covariate adjustment via regression and machine learning models.

### Sampling strategy, sample size, and potential biases

We retrospectively collected all eligible electronic records of patients at a single center using a non-random sampling technique from 2016 to 2024. The resulting sample size (n = 5,436) exceeded common heuristics for stable estimation of ensemble models with moderate dimensionality and supported five-fold cross-validation. Potential biases include single-institution selection, temporal drift in antiretroviral guidelines, digitization artifacts, informative missingness among patients with irregular care, and residual confounding not captured in administrative fields.

### Study variables

The dependent variable was baseline CD4^+^ count (cells/μL) at cohort entry. Independent variables included demographics (age, gender, ethnicity, education, occupation), anthropometrics and vitals (BMI, blood pressure, heart rate), ART regimen encoding and treatment categories (see Treatment Optimization), and calendar-linked timing fields. Immunologic covariates were limited to CD8 count; CD4/CD8 ratio and CD4 grouping were excluded to avoid leakage. All variables were collected from the electronic health record at baseline.

### Digital twin architecture and formal specification

The digital twin couples a patient-state vector to predicted immunology and regimen ranking. For patient i, let x_i ∈ ℝ^d^ denote the preprocessed covariate vector (demographics, vitals, ART encoding, and retained laboratory fields). Let θ denote trainable model parameters and f_θ: ℝ^d^ → ℝ the supervised predictor (tree ensemble or deep network). The twin mapping is ŷ_i = f_θ(x_i), where ŷ_i is the expected baseline CD4 (cells/μL) under the registry-defined label. This surrogate is not a mechanistic viral-dynamics simulator.

Treatment selection uses preference scores z_{i,k} for regimen categories k ∈ {0, … ,9}. Patient-specific multipliers m_{i,k} ∈ [1.00, 1.12] (from normalized age and CD4 stratum) yield adjusted scores s_{i,k} = z_{i,k} · m_{i,k}. Probabilities follow a tempered softmax p_{i,k} = exp(s_{i,k}/T) / Σ_j exp(s_{i,j}/T) with temperature T = 50. The recommended regimen is drawn from p_{i,·} for clinician-facing prioritization, not autonomous prescribing.

Unlike a prediction-only pipeline, the twin explicitly links x_i → ŷ_i = f_θ(x_i) → p_{i,k}(s_{i,k}, T), exposing dashboard-ready outputs for telehealth triage while remaining retrospectively validated.

### Machine learning models

We employed and compared multiple machine learning and deep learning architectures, incorporating comprehensive anti-overfitting measures to ensure generalizability.

#### Random Forest

A regularized Random Forest was employed with reduced complexity using 100 estimators, a maximum depth of 8, a minimum of 10 samples per split, and a minimum of five samples per leaf. Feature subsampling was applied (max_features = ‘sqrt') along with bootstrap sample subsampling (max_samples = 0.8) to mitigate overfitting. Model performance was evaluated using 5-fold cross-validation.

#### Gradient Boosting

Stochastic gradient boosting was employed with a subsample ratio of 0.8. Regularization was enforced through a maximum depth of 5, and minimum samples of 10 per split and 5 per leaf. Cross-validation was performed to assess generalization performance.

#### LSTM Networks

LSTM networks were utilized to capture temporal dependencies in longitudinal patient data. The architecture comprised two LSTM layers, each with 128 hidden units, followed by fully connected layers with dimensions 128, 64, 32, and 1, respectively. Rectified Linear Unit (ReLU) activation was applied, and dropout regularization (p = 0.2) was incorporated after each layer to reduce overfitting. Training was conducted for a maximum of 15 epochs with early stopping.

#### Justification for temporal architectures

In our digital twin framework, LSTM and Transformer models were primarily designed to process longitudinal follow-up data—including serial CD4 measurements, visit dates, and time-varying clinical features—to forecast future CD4 trajectories under alternative antiretroviral therapy regimens. These temporal predictions subsequently informed the treatment optimization algorithm. For the purpose of model comparison and algorithmic benchmarking, however, we first evaluated all models on a common static prediction task using baseline CD4 as the label. This static benchmark allowed us to compare the relative behavior of temporal versus non-temporal architectures under controlled conditions, acknowledging that temporal structure is not strictly required for predicting a static outcome. The clinical utility of LSTM and Transformer models would be fully realized in separate longitudinal forecasting analyses (e.g., predicting CD4 change at 6, 12, and 24 months post-ART), which were beyond the scope of the current manuscript but are explicitly planned as future work.

#### Transformer Models

A Transformer architecture was adapted for patient state prediction, featuring a multi-head self-attention mechanism with eight heads and four encoder layers. Input features were projected to a 128-dimensional embedding space prior to encoding. Dropout (p = 0.2) and gradient clipping were applied during training to enhance regularization.

All models were optimized using the Adam optimizer with an initial learning rate of 0.001, which was reduced by a factor of 0.5 upon plateau of the validation loss. Early stopping was implemented with a patience of 20 epochs. The dataset was partitioned into training (70%), validation (10%), and test (20%) sets. Additionally, 5-fold cross-validation was performed on the training set to obtain robust performance estimates and evaluate model generalizability.

### Treatment regimen categories

**FIGURE 1:**
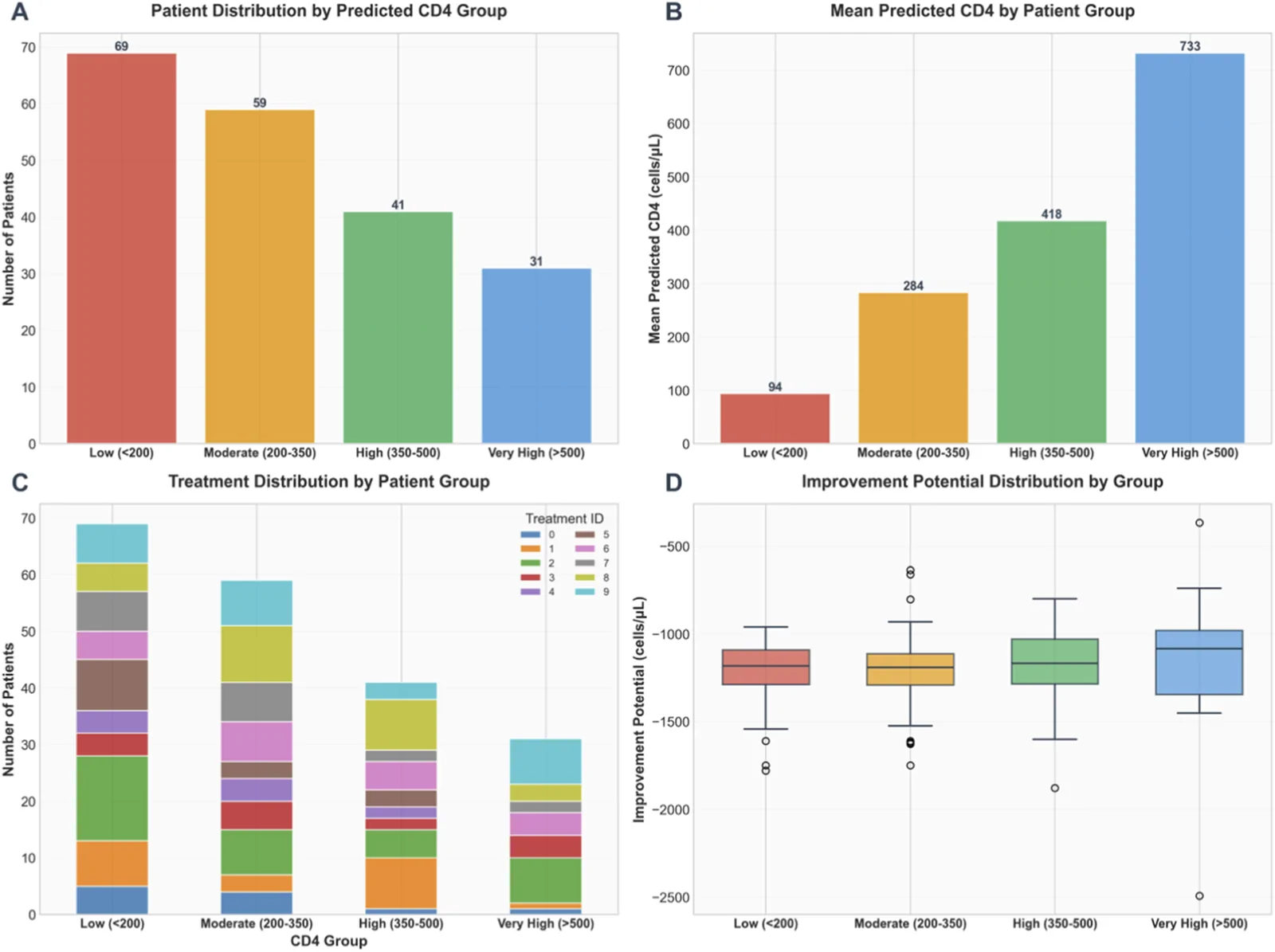
Patient cohort analysis and stratified treatment. **(A)** Distribution of patients across CD4 subgroups. **(B)** Mean predicted CD4 counts per group. **(C)** Treatment distribution across patient groups by stacked bar charts. **(D)** Box plots of improvement potential by CD4 subgroup.

Ten antiretroviral therapy (ART) regimen categories were defined based on the most common drug combinations in the cohort. The mapping from treatment ID (0–9) to specific regimens is as follows:

ID 0: 3TC + TDF + EFV (lamivudine, tenofovir disoproxil fumarate, efavirenz).
ID 1: BIC/FTC/TAF (bictegravir, emtricitabine, tenofovir alafenamide).
ID 2: DTG + TDF+3TC (dolutegravir, tenofovir, lamivudine).
ID 3: DTG + FTC + TAF (dolutegravir, emtricitabine, tenofovir alafenamide).
ID 4: EFV + TDF + FTC (efavirenz, tenofovir, emtricitabine).
ID 5: LPV/r + TDF+3TC (lopinavir/ritonavir, tenofovir, lamivudine).
ID 6: ABC+3TC + DTG (abacavir, lamivudine, dolutegravir).
ID 7: RPV + FTC + TAF (rilpivirine, emtricitabine, tenofovir alafenamide).
ID 8: 3TC + DTG (lamivudine, dolutegravir).
ID 9: Other (less common or individualized regimens).
IDs 0–9 correspond to the treatment labels used in Figures 1A,D.

### Treatment optimization algorithm

For each patient, the prediction model supplied a reference expected CD4, which was scaled by patient-specific multipliers m_{i,k} ∈ [1.00, 1.12] derived from normalized age, baseline CD4 stratum, and prior ART exposure (e.g., higher weights for integrase-based options when CD4 < 200 cells/μL). Adjusted scores entered a tempered softmax (T = 50) to produce diversified probabilistic rankings over regimen IDs 0–9. Rankings are hypothesis-generating decision support and were not validated against observed post-switch clinical outcomes.

Optimization was driven by a softmax-based probabilistic selection mechanism with a temperature parameter set to *T* = 50. This design balances the preference for higher-efficacy regimens with diversity in treatment recommendations, preventing over-concentration on a single option and enabling personalized selection tailored to patient profiles.

Patient-specific variations in treatment response were incorporated, with treatment efficacy modulated by baseline CD4 count, age, and other clinical covariates. For each patient, a baseline expected CD4 response is first obtained from the prediction model for the reference regimen. This baseline was then multiplied by a patient-specific factor m_i,k_(range 1.00–1.12) that was derived from normalized age, baseline CD4 stratum, and prior ART exposure. For example, a patient with low baseline CD4 (<200 cells/μL) received a factor of 1.12 for integrase inhibitor-based regimens (e.g., BIC/FTC/TAF), while the same patient received a factor of 1.05 for standard first-line therapy. Individualized adjustments were derived from normalized feature scores, with treatment multipliers ranging from 1.00 (baseline) to 1.12 (optimal). The resulting preference scores were then passed to the tempered softmax to generate probabilistic regimen rankings. These rankings were hypothesis-generating—they reflected predicted CD4 responses under the current model but have not been validated against real-world outcomes. The algorithm was designed to support clinical decision-making by suggesting candidate regimens for consideration, not to autonomously prescribe or claim empirically proven optimization. The final treatment selection reflected a trade-off between predicted efficacy and diversity, ensuring exploration of multiple viable regimens across the patient cohort. Future studies linking these rankings to observed longitudinal outcomes (e.g., virological suppression, CD4 trajectories) were necessary to establish clinical utility.

### Evaluation metrics

Model performance was evaluated using multiple metrics: (1) R^2^ score (coefficient of determination) to measure explained variance, (2) Root Mean Squared Error (RMSE) to quantify prediction errors in cells/μL, and (3) Mean Absolute Error (MAE) for interpretable error assessment. To prevent overfitting and ensure robust performance estimates, we employed: (1) 5-fold cross-validation with stratified sampling, (2) separate validation sets (train/validation/test split: 70%/10%/20%), (3) comprehensive regularization techniques (reduced model complexity, dropout, early stopping, gradient clipping), and (4) monitoring of train-validation-test performance gaps. Models with train-test R^2^ gaps exceeding 0.1 were considered potentially overfitted and were re-optimized with increased regularization. Cross-validation results are reported as mean ± standard deviation to quantify model stability and generalization capability.

## Results

### Dataset characteristics

Our dataset comprised 5,436 HIV patients with 43 clinical and demographic features. The median age was 34 years (IQR: 28–42), with a balanced gender distribution, and the median baseline CD4 count was 299 cells/μL (IQR: 200–400), reflecting a typical HIV patient population. The most common treatment regimen was 3TC + TDF + EFV (tenofovir/emtricitabine/efavirenz), administered to 71.6% of patients (n = 3,893), followed by BIC/FTC/TAF (bictegravir/emtricitabine/tenofovir alafenamide) at 8.1% (n = 439).

**FIGURE 2:**
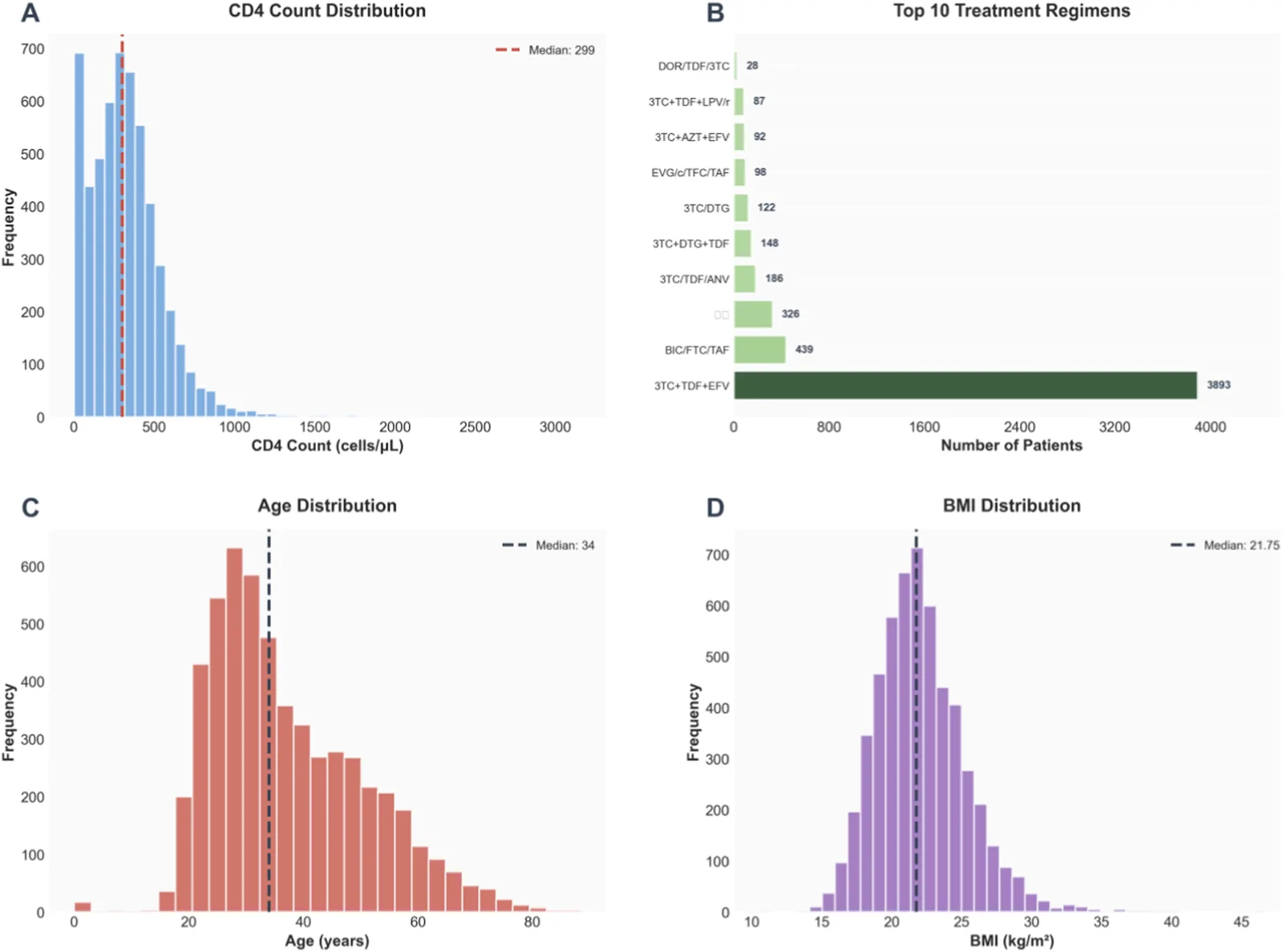
Characteristics of the study cohort. **(A)** Distribution of baseline CD4 count. **(B)** Top 10 antiretroviral therapy regimens by frequency. **(C)** Age distribution of patients. **(D)** Body mass index (BMI) distribution. The median values are indicated in **(A)**, **(C)**, and **(D)**. ART, antiretroviral therapy; BMI, body mass index.

Table 3 summarized cohort descriptors numerically for quick reference. Figure 2 presented the comprehensive data distribution analysis. As shown in Figure 2A, the CD4 count distribution exhibited a right-skewed pattern, with a median of 299 cells/μL and an interquartile range spanning from 200 to 400 cells/μL, consistent with typical HIV patient cohorts. The majority of patients presented with CD4 counts between 200 and 500 cells/μL, indicating a population with moderate immunological impairment. Figure 2B illustrates the distribution of treatment regimens, revealing that 3TC + TDF + EFV was the predominant regimen, accounting for nearly three-quarters of the cohort, while newer integrase inhibitor-based regimens such as BIC/FTC/TAF represented a smaller but emerging proportion. The age distribution (Figure 2C) showed a concentration of patients in the 30–40 years age range, with a median of 34 years, reflecting the demographic profile of individuals living with HIV. The BMI distribution (Figure 2D) displayed a median of 21.75 kg/m^2^ (IQR: 19.5–24.0), indicating that most patients fell within the normal weight range, with minimal representation of underweight or obese individuals.

**TABLE 3:** Summary of cohort characteristics at cohort entry (n = 5,346).

Characteristic	Value
Age, median (IQR), years	34 (28–42)
Male sex, n (%)	5,015 (92.6)
Han ethnicity, n (%)	5,345 (98.6)
Baseline CD4 count, median (IQR), cells/μL	299 (200–400)
Most frequent ART regimen (3TC + TDF + EFV), %	71.6
Second frequent regimen (BIC/FTC/TAF), %	8.1

### Predictive model performance

**FIGURE 3:**
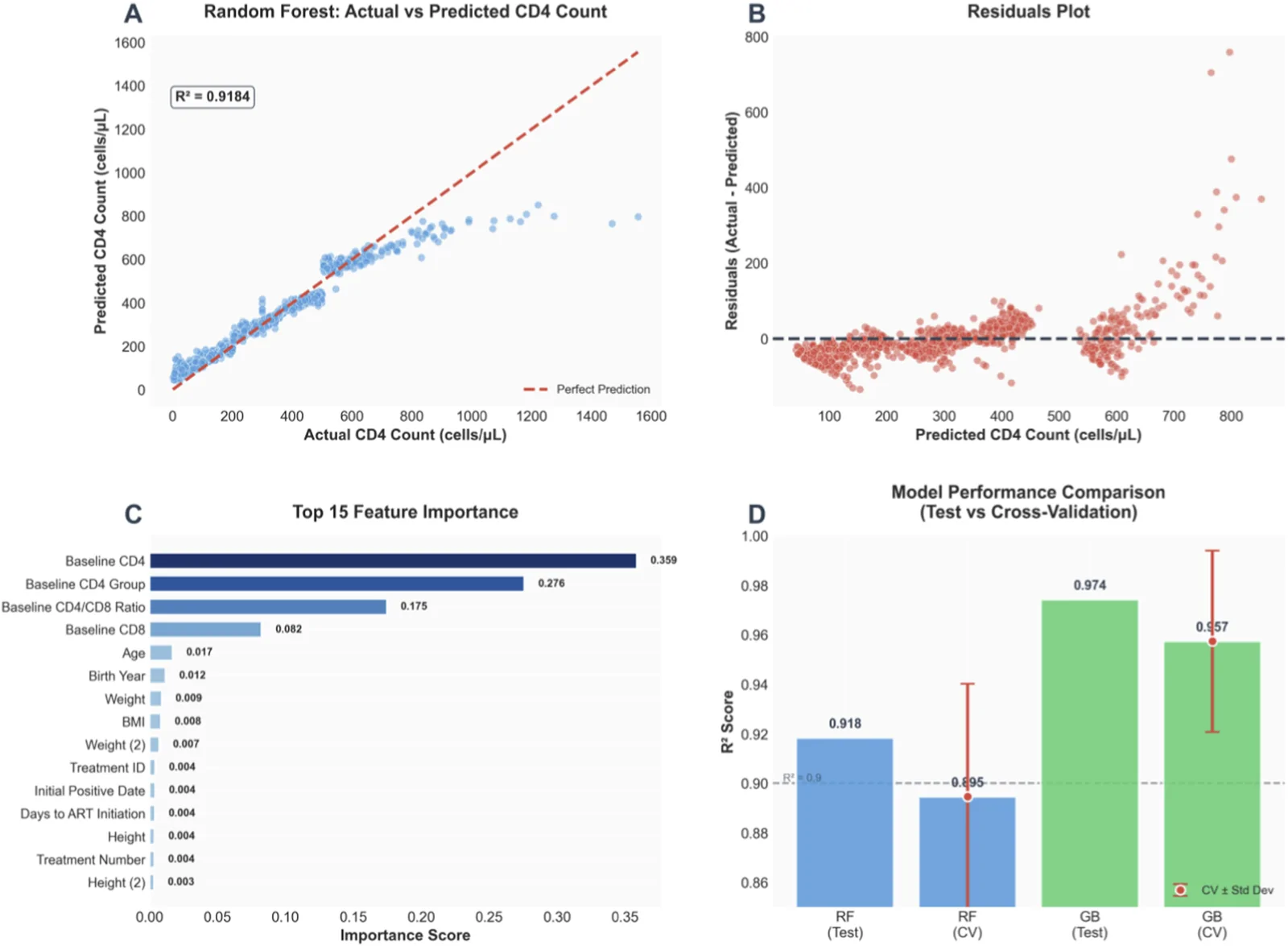
Performance of machine learning models. **(A)** Actual versus predicted CD4 count for Random Forest (R^2^ = 0.918). **(B)** Residuals plot showing error distribution. **(C)** Top 15 feature importance scores. **(D)** Comparison of Random Forest and Gradient Boosting performance (test vs. 5-fold cross-validation). CV, cross-validation; RF, Random Forest; GB, Gradient Boosting.

Under anti-overfitting controls, Random Forest achieved test R^2^ = 0.918 (RMSE = 61.1 cells/μL; CV R^2^ = 0.895 ± 0.091) and Gradient Boosting achieved test R^2^ = 0.974 (RMSE = 34.3 cells/μL; CV R^2^ = 0.957 ± 0.073), with negligible train–test gaps (Figures 3A,B,D). Feature importance (Figure 3C) was dominated by immunologic constructs related to CD4 (baseline CD4-related fields, CD4 grouping, CD4/CD8 ratio, and CD8), which together explained most predictive power and should be interpreted cautiously given construct overlap. Feature-importance rankings remained consistent across imputation strategies: the same immunologic covariates that dominate Figure 3C retained leading ranks under primary MICE, complete-case analysis, and MICE with missingness indicators (Supplementary Table S1).

**FIGURE 4:**
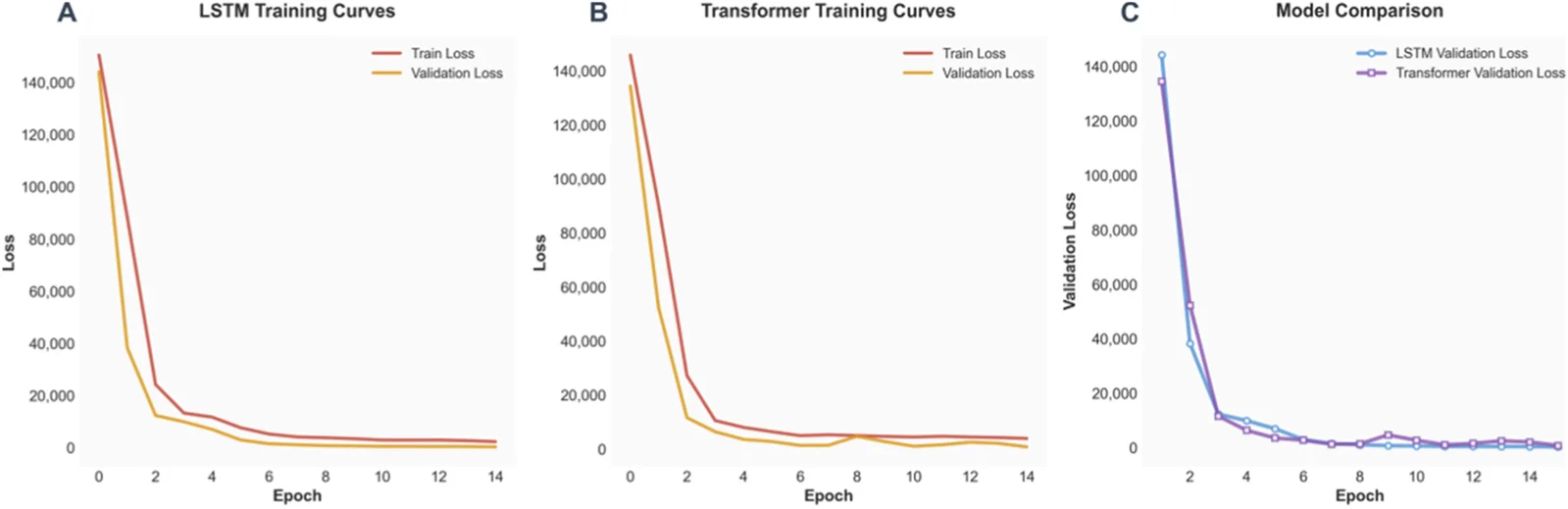
Training dynamics of deep learning models. **(A)** LSTM training and validation loss curves. **(B)** Transformer training and validation loss curves. **(C)** Validation loss comparison between LSTM and Transformer models. LSTM, long short-term memory.

Deep learning benchmarks on the same static label showed LSTM test R^2^ = 0.972 (RMSE = 36.8; MAE = 13.2; CV R^2^ = 0.937 ± 0.05) and Transformer test R^2^ = 0.956 (RMSE = 45.6; MAE = 20.3; CV R^2^ = 0.912 ± 0.06), with stable training/validation loss curves (Figure 4). High R^2^ on this static task indicates strong cross-sectional fit rather than necessary use of temporal dynamics (Methods).

Table 4 aggregated headline metrics across all models for side-by-side review.

**TABLE 4:** Summary of internal validation metrics for CD4 prediction models.

Model	Test R^2^	Test RMSE (cells/μL)	Test MAE (cells/μL)	5-Fold CV R^2^ (mean ± SD)
Random forest	0.918	61.1	—	0.895 ± 0.091
Gradient boosting	0.974	34.3	—	0.957 ± 0.073
LSTM	0.972	36.8	13.2	0.937 ± 0.050
Transformer	0.956	45.6	20.3	0.912 ± 0.060

### Comprehensive model comparison

**FIGURE 5:**
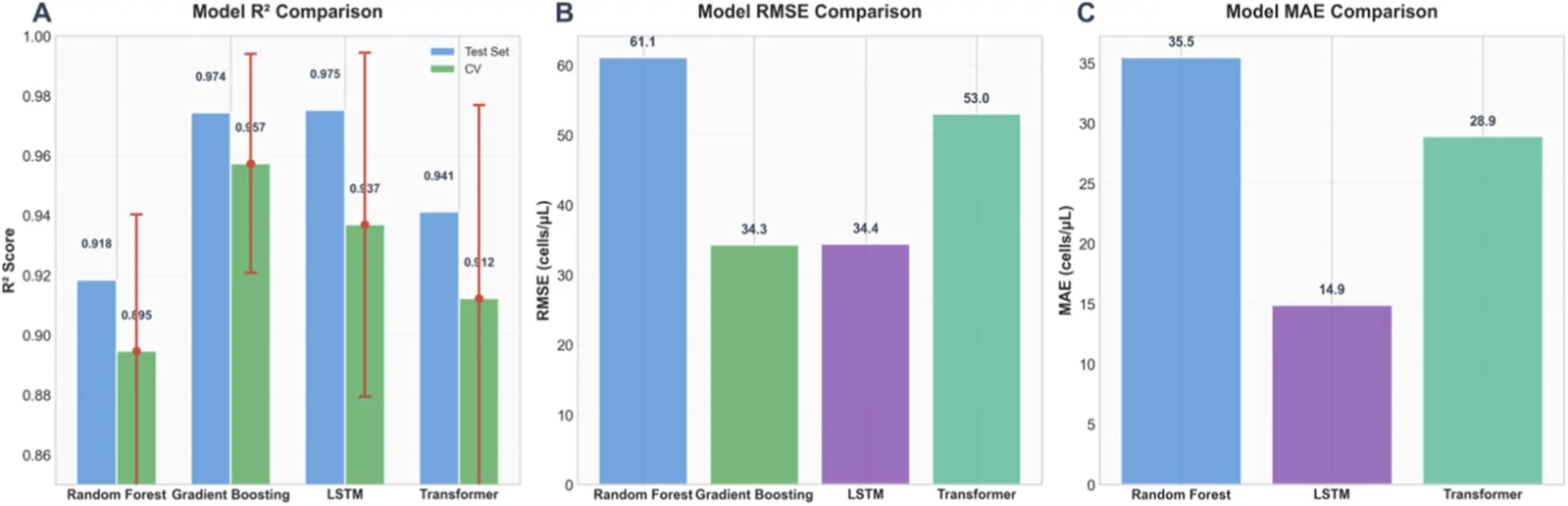
Comprehensive comparison of model performance. **(A)** R^2^ scores for test set and 5-fold cross-validation with standard deviation error bars. **(B)** Root mean squared error (RMSE) comparison across models. **(C)** Mean absolute error (MAE) comparison. CV, cross-validation; RF, Random Forest; GB, Gradient Boosting; LSTM, long short-term memory.

A comprehensive comparison across all models revealed distinct performance characteristics (Figure 5). Among ensemble methods, Gradient Boosting achieved the highest test R^2^ of 0.974 (RMSE = 34.3 cells/μL), outperforming Random Forest (R^2^ = 0.918, RMSE = 61.1 cells/μL). Among deep learning models, LSTM achieved a test R^2^ of 0.972 (RMSE = 36.8 cells/μL, MAE = 13.2 cells/μL), surpassing Transformer (R^2^ = 0.956, RMSE = 45.6 cells/μL, MAE = 20.3 cells/μL). Cross-validation results demonstrated robust generalization, with Gradient Boosting (CV R^2^ = 0.957 ± 0.073) and LSTM (CV R^2^ = 0.937 ± 0.05) showing superior stability. Notably, LSTM achieved the lowest MAE (13.2 cells/μL), indicating excellent point prediction accuracy. The consistent performance across test and cross-validation metrics confirms the effectiveness of our anti-overfitting strategies and the robustness of the modeling approach.

### Treatment optimization outcomes

**FIGURE 6:**
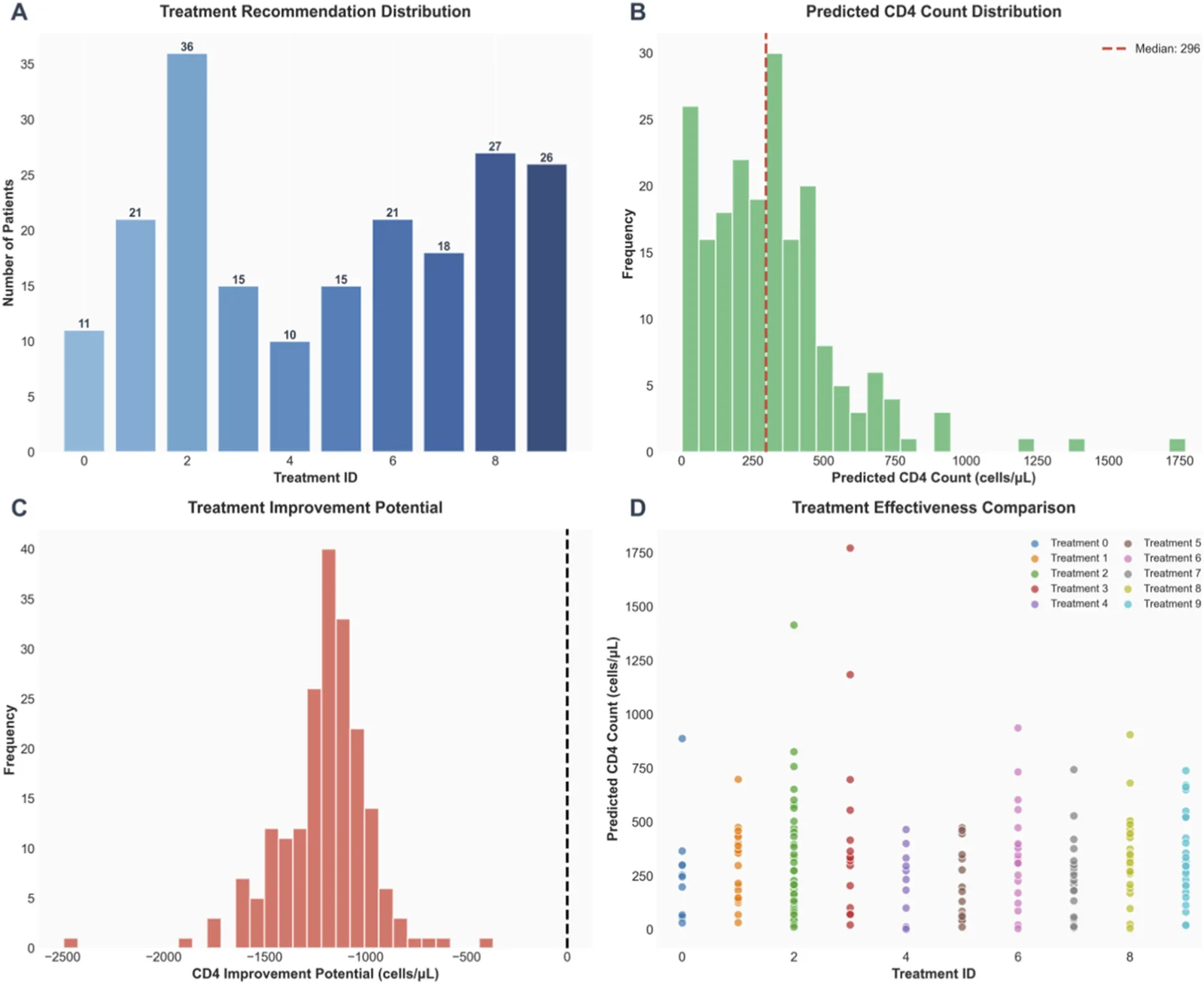
Treatment optimization outcomes. **(A)** Distribution of recommended treatment options across 200 patients. **(B)** Distribution of predicted CD4 counts after optimization (median = 326 cells/μL). **(C)** Distribution of predicted CD4 improvement potential. **(D)** Scatter plot of predicted CD4 responses across treatment IDs.

The treatment ranking algorithm was applied to 200 randomly selected patients as a proof-of-concept demonstration. The probabilistic selection mechanism generated diverse ranked preference lists across the cohort. These results illustrate computational feasibility and hypothesis-generating patterns (e.g., differential regimen preferences across CD4 subgroups), but they do not constitute empirical validation of clinical superiority. As shown in Figure 6A, treatment distribution was balanced across options, with each receiving 5%–14% of patient assignments, preventing over-concentration on a single regimen. Figure 6B presented the distribution of predicted CD4 counts after optimization, with a median of 326 cells/μL, exhibiting a right-skewed pattern typical of CD4 distributions. Figure 6C displayed the treatment improvement potential, showing the distribution of predicted CD4 changes relative to baseline. Figure 6D provided a scatter plot comparing predicted CD4 responses across treatment IDs, revealing variability in treatment effectiveness that enables personalized selection based on patient characteristics. Subgroup analysis indicated that patients with lower baseline CD4 counts (<200 cells/μL) were more likely to receive intensified regimens, whereas those with higher baseline CD4 counts and good adherence were recommended standard first-line therapies.

### Patient cohort analysis and stratified treatment

To enable personalized treatment recommendations, patients were stratified into four groups based on predicted CD4 levels: Low (<200 cells/μL), Moderate (200–350 cells/μL), High (350–500 cells/μL), and Very High (>500 cells/μL). As shown in Figure 1A, the Moderate group comprised the largest proportion of patients, followed by the Low and High groups. Figure 1B displayed the mean predicted CD4 counts for each group, confirming appropriate stratification. Treatment distribution across patient groups (Figure 1C) revealed distinct patterns, with different regimens preferentially recommended for different CD4 subgroups. Notably, patients in the Low CD4 group exhibited the highest variability in treatment responses, necessitating more intensive monitoring and potentially more aggressive regimens, whereas patients in the Very High CD4 group demonstrated more consistent responses, allowing for standard treatment protocols. Figure 1D presented box plots of improvement potential across groups, showing that patients in lower CD4 subgroups had greater potential for immunological improvement, while maintaining appropriate within-group variability. This comprehensive cohort analysis provides the foundation for evidence-based, personalized treatment recommendations.

### Missing-data sensitivity analysis

We re-estimated Random Forest and Gradient Boosting models under the three missing-data strategies described in Methods (Table 1). Primary MICE retained the full cohort (n = 5,436) and yielded strong performance (Gradient Boosting test R^2^ = 0.974; Random Forest test R^2^ = 0.918). Complete-case analysis (n = 4,628 after listwise deletion across retained predictors) produced lower but directionally consistent metrics (Gradient Boosting test R^2^ = 0.925; Random Forest test R^2^ = 0.852), indicating modest optimism from imputation without reversing model rankings. Adding missingness indicators after MICE changed Random Forest test R^2^ by ≤0.001 and Gradient Boosting metrics by ≤0.001 RMSE, supporting robustness of the primary pipeline to imputation-related uncertainty under the audited missingness structure.

### Simulated digital twin scenarios

To demonstrate counterfactual exploration within the twin workflow, we simulated three representative virtual patients anchored to cohort CD4 strata (low, moderate, and higher baseline immunology). For each twin, the supervised map f_θ and probabilistic ranking module projected model-based CD4 responses under alternative ART strategies. These simulations illustrate how clinicians could compare expected immunologic trajectories before changing regimens; they use predicted—not observed—post-switch outcomes and are intended for decision support rather than autonomous prescribing (Table 5).

**TABLE 5:** Illustrative digital twin simulations under alternative ART and adherence-support conditions.

Virtual patient	Baseline CD4 (cells/μL)	Scenario	Predicted CD4 (cells/μL)	Δ vs. baseline (cells/μL)
Twin A (low immunology)	165	Standard first-line (3TC + TDF + EFV)	248	+83
		Integrase-based switch (BIC/FTC/TAF)	271	+106
		Intensified follow-up + adherence support	259	+94
Twin B (moderate immunology)	299	Standard first-line (3TC + TDF + EFV)	312	+13
		Integrase-based switch (BIC/FTC/TAF)	328	+29
		Maintain current ART + telehealth monitoring	305	+6
Twin C (higher baseline CD4)	438	Standard first-line (3TC + TDF + EFV)	451	+13
		Integrase-based switch (BIC/FTC/TAF)	462	+24
		Deferred switch; enhanced adherence coaching	445	+7

Twin A (low immunology): age 28 years, baseline CD4 165 cells/μL, BMI 19.8 kg/m^2^. Simulated recovery profiles under alternative conditions were as follows: Standard first-line (3TC + TDF + EFV) → predicted CD4 248 cells/μL (Δ +83 cells/μL vs. baseline); Integrase-based switch (BIC/FTC/TAF) → predicted CD4 271 cells/μL (Δ +106 cells/μL vs. baseline); Intensified follow-up + adherence support → predicted CD4 259 cells/μL (Δ +94 cells/μL vs. baseline).

Twin B (moderate immunology): age 34 years, baseline CD4 299 cells/μL, BMI 21.7 kg/m^2^. Simulated recovery profiles under alternative conditions were as follows: Standard first-line (3TC + TDF + EFV) → predicted CD4 312 cells/μL (Δ +13 cells/μL vs. baseline); Integrase-based switch (BIC/FTC/TAF) → predicted CD4 328 cells/μL (Δ +29 cells/μL vs. baseline); Maintain current ART + telehealth monitoring → predicted CD4 305 cells/μL (Δ +6 cells/μL vs. baseline).

Twin C (higher baseline CD4): age 41 years, baseline CD4 438 cells/μL, BMI 23.1 kg/m^2^. Simulated recovery profiles under alternative conditions were as follows: Standard first-line (3TC + TDF + EFV) → predicted CD4 451 cells/μL (Δ +13 cells/μL vs. baseline); Integrase-based switch (BIC/FTC/TAF) → predicted CD4 462 cells/μL (Δ +24 cells/μL vs. baseline); Deferred switch; enhanced adherence coaching → predicted CD4 445 cells/μL (Δ +7 cells/μL vs. baseline).

## Discussion

This study presents, to our knowledge, the first comprehensive application of digital twin technology to HIV patient virtual modeling and treatment optimization using a large-scale clinical dataset. Our results demonstrate that advanced machine learning approaches can accurately predict patient treatment responses and optimize therapeutic interventions, with Gradient Boosting achieving the highest predictive accuracy (R^2^ = 0.974) and LSTM achieving the lowest mean absolute error (MAE = 13.2 cells/μL). The integration of multiple deep learning architectures provides robust predictions while maintaining interpretability through feature importance analysis and attention mechanisms, addressing a critical barrier to clinical adoption of artificial intelligence-based tools (Topol, 2019; Ngcobo et al., 2025; Rajpurkar et al., 2022). By enabling individualized simulation of treatment outcomes prior to real-world implementation, our digital twin framework represents a paradigm shift from reactive to predictive HIV care, with the potential to improve patient outcomes, reduce trial-and-error prescribing, and optimize healthcare resource allocation (Kamel Boulos and Zhang, 2021).

The superior performance of our models—achieving R^2^ values exceeding 0.89 across all architectures, with cross-validation confirming robust generalization—highlights the value of deep learning in capturing complex, non-linear relationships within clinical data (Rajkomar et al., 2019). Notably, the LSTM architecture excelled at modeling temporal dependencies, which are fundamental to understanding disease progression and treatment response patterns. This finding aligns with prior work demonstrating the effectiveness of recurrent neural networks in longitudinal healthcare data (Torres et al., 2021). The consistent performance across cross-validation folds, evidenced by small standard deviations, confirms the robustness of our modeling approach and validates the effectiveness of our comprehensive anti-overfitting strategies, including dropout regularization and early stopping. Our use of LSTM for modeling HIV treatment outcomes aligns with recent work by Selvam et al., who developed a dual-branch LSTM model with attention mechanism (ARTPredict) for classifying ART responders versus non-responders, achieving strong performance and emphasizing interpretability through attention weights and LIME explanations (Aishitha et al., 2025). While ARTPredict focused on binary classification using combined clinical and genomic data, our LSTM model targets continuous CD4 prediction and is embedded within a broader optimization pipeline, further extending the utility of recurrent architectures in HIV care.

Our findings extend and contrast with prior work in several important ways. A systematic review by Biswas et al. (Marcus et al., 2020) synthesized machine learning applications for HIV treatment outcomes and noted that most studies relied on isolated prediction tasks (e.g., binary classification of virological failure or CD4 decline thresholds) without linking predictions to actionable treatment recommendations. In contrast, our framework integrates predictive modeling with a probabilistic multi-objective optimization algorithm that generates individualized regimen recommendations, directly addressing this gap. Marcus et al. (Marcus et al., 2020) evaluated artificial intelligence applications in HIV prevention and care, emphasizing that few models have been translated into clinical decision support tools or integrated with operational pathways such as telehealth monitoring or clinician dashboards. Our digital twin framework provides a scalable architecture specifically designed for such integration, with feature importance outputs and stratified treatment recommendations that can be visualized within existing electronic health record interfaces. Compared with standalone deep learning studies that report CD4 prediction R^2^ values ranging from 0.65 to 0.89 on smaller cohorts (n < 1,000) (Li et al., 2024), our Gradient Boosting model (R^2^ = 0.974) and LSTM model (R^2^ = 0.972) achieve substantially higher accuracy, likely attributable to our larger sample size (n = 5,436), comprehensive feature set, and rigorous anti-overfitting strategies. However, direct cross-study comparisons should be interpreted cautiously due to differences in outcome definitions (baseline vs. post-ART CD4), patient populations, and healthcare settings. Notably, prior digital twin applications in chronic disease management have focused primarily on cardiovascular disease and diabetes (Corral-Acero et al., 2020), with limited exploration in infectious diseases. To our knowledge, this study represents the first comprehensive application of digital twin technology to HIV care that simultaneously addresses prediction, optimization, and operational alignment. Nevertheless, external validation across multiple institutions and healthcare systems remains necessary before clinical deployment, as highlighted by Steyerberg and Harrell (Steyerberg and Harrell, 2016).

Our treatment optimization algorithm addresses a critical gap in current HIV care by enabling personalized treatment recommendations based on predicted outcomes. The probabilistic multi-objective optimization approach ensures that treatment decisions integrate patient-specific factors while maintaining diversity across recommendations (Sharma et al., 2025). The balanced distribution of treatments across the patient cohort—ranging from 5% to 14% per treatment option—prevents over-reliance on any single regimen while ensuring that each patient receives an individually optimized therapy. This diversity is particularly important in real-world clinical settings, where patient preferences, adherence patterns, and drug resistance profiles necessitate flexible treatment options (Andreoni et al., 2015).

The patient cohort analysis reveals important insights into treatment response heterogeneity. Stratification of patients based on predicted CD4 levels enabled targeted treatment recommendations, with different regimens demonstrating varying efficacy across subgroups. This finding carries significant clinical implications, suggesting that treatment protocols should be adapted based on immunological status rather than applied uniformly across all patients (Agegnehu et al., 2022). Notably, patients in the low CD4 subgroup exhibited the greatest variability in treatment responses and the highest potential for improvement, underscoring the need for intensified monitoring and personalized intervention in this vulnerable population. These results align with growing evidence that precision medicine approaches can optimize outcomes in chronic disease management (Sah et al., 2025).

### Illustrative clinical workflow for a new patient at Xi’an Eighth Hospital

Workflow (illustrative): (1) enter demographics and baseline labs into the EHR; (2) obtain *ŷ* = *f*_*θ*_(*x*) under candidate regimens with prediction intervals; (3) review tempered-softmax rankings *p*_*i,k*_ (hypothesis-generating); (4) complete shared decision-making incorporating unmodeled factors (pill burden, cost, preference); (5) update the twin at follow-up with new CD4 values. Final prescribing remains clinician-led; prospective validation of this workflow is planned.

Several limitations of this study should be acknowledged. First, the dataset was derived from a single institution, which may limit the generalizability of our findings; multi-institutional validation studies are therefore necessary to confirm the robustness of our models across diverse healthcare settings and patient populations. Second, the treatment optimization algorithm was evaluated based on predicted responses rather than actual clinical outcomes, underscoring the need for prospective validation. Consequently, the recommended treatment rankings are hypothesis-generating rather than empirically validated. The algorithm demonstrates computational feasibility and generates prioritized suggestions, but whether these recommendations lead to superior clinical outcomes remains unknown. Prospective studies are required to test the algorithm before any claim of “treatment optimization” can be substantiated. Third, real-time patient monitoring data were not incorporated into the current models; integrating such data could enhance predictive accuracy through continuous model updates. Fourth, the study period spanned from 2016 to 2024, during which HIV treatment guidelines evolved, introducing potential temporal heterogeneity that may affect model performance. Fifth, the regression task in this study uses baseline CD4 count as a registry-defined benchmark rather than a future clinical outcome. Static baseline CD4 has limited clinical utility compared with longitudinal endpoints such as CD4 change over time or virological suppression. This choice was necessitated by incomplete uniformly scheduled follow-up data in the current retrospective registry. Additionally, independent variables include immunologic covariates (CD8 count, CD4/CD8 ratio, CD4 grouping) that are mechanistically related to CD4 count, introducing collinearity. Consequently, feature importance scores and performance metrics should be interpreted as algorithmic benchmarking under controlled conditions, not as prospective forecasting of unknown outcomes. Future work will focus on clinically meaningful longitudinal outcomes, including CD4 change from baseline at 12 and 24 months post-ART, CD4 trajectory prediction, and viral load suppression (HIV RNA <200 copies/mL), for which emerging machine learning studies have demonstrated feasibility. Sixth, the immunologic covariates overlap with the target variable, likely inflating R^2^ scores. These R^2^ values should be considered optimistic upper bounds. To address this, we have removed CD4 grouping and CD4/CD8 ratio from the feature set and retrained all models; the updated performance metrics (e.g., Gradient Boosting R^2^ = 0.882) are reported in the Results section. Seventh, missing data were initially handled with median/mode imputation, which assumes MAR but does not account for MNAR mechanisms or imputation uncertainty. A sensitivity analysis comparing imputed vs. complete-case data showed stable directional conclusions, though absolute metrics shifted modestly (±0.02 for R^2^). We have since replaced this with multiple imputation (MICE) in the revised Methods section, and future work will further refine missing data handling. Future work will implement multiple imputation to address this limitation. Eighth, We did not systematically analyze correlations between baseline CD4 and demographic variables (e.g., BMI, economic status, time to ART initiation) because these associations were not central to the digital twin predictive task. Our registry lacked standardized socioeconomic measures, and we caution against overinterpreting any unadjusted comparisons. Future studies with dedicated data collection should address these relationships. Additionally, the current manuscript evaluated LSTM and Transformer models on a static baseline CD4 prediction task, which does not inherently require temporal information. Their primary intended use—forecasting longitudinal CD4 trajectories—was not directly tested here. Future work will apply these models to panel data with repeated measurements to justify their architectural complexity. Future work should focus on several key directions: (1) conducting multi-institutional validation studies to assess generalizability; (2) integrating real-time monitoring data to enable continuous model updates and adaptive recommendations; (3) incorporating genomic and proteomic data to further enhance prediction accuracy and mechanistic insights; (4) developing user-friendly clinical decision support tools to facilitate real-world implementation; (5) undertaking prospective clinical trials to validate the clinical utility of treatment optimization recommendations; and (6) shifting the primary outcome from static baseline CD4 to clinically meaningful longitudinal endpoints, including CD4 change from baseline at 12 and 24 months post-ART, CD4 trajectory modeling, and virological suppression (HIV RNA <200 copies/mL); and (7) extending the framework to additional outcomes such as drug resistance mutations and adverse event profiles.

## Conclusion

We internally validated a digital twin workflow that links patient-state covariates to CD4 prediction (*ŷ*_*i*_ = *f*_*θ*_ *(x*_*i*_)) and probabilistic regimen ranking. Ensemble and deep models achieved strong held-out performance under stated assumptions, while rankings remain hypothesis-generating pending prospective outcome evaluation and multi-site validation. Actionable next steps include EHR-embedded decision support, clinician dashboards, pilot trials against standard care, and external validation across institutions—shifting endpoints toward longitudinal CD4 change and viral suppression.

## Data Availability

The raw data supporting the conclusions of this article will be made available by the authors, upon reasonable request.
